# Coordination in Fast Repetitive Violin-Bowing Patterns

**DOI:** 10.1371/journal.pone.0106615

**Published:** 2014-09-10

**Authors:** Erwin Schoonderwaldt, Eckart Altenmüller

**Affiliations:** Institute of Music Physiology and Musicians' Medicine, Hanover University of Music, Drama and Media, Hanover, Germany; University of California, Merced, United States of America

## Abstract

We present a study of coordination behavior in complex violin-bowing patterns involving simultaneous bow changes (reversal of bowing direction) and string crossings (changing from one string to another). Twenty-two violinists (8 advanced amateurs, 8 students with violin as major subject, and 6 elite professionals) participated in the experiment. We investigated the influence of a variety of performance conditions (specific bowing patterns, dynamic level, tempo, and transposition) and level of expertise on coordination behavior (a.o., relative phase and amplitude) and stability. It was found that the general coordination behavior was highly consistent, characterized by a systematic phase lead of bow inclination over bow velocity of about 15° (i.e., string crossings were consistently timed earlier than bow changes). Within similar conditions, a high individual consistency was found, whereas the inter-individual agreement was considerably less. Furthermore, systematic influences of performance conditions on coordination behavior and stability were found, which could be partly explained in terms of particular performance constraints. Concerning level of expertise, only subtle differences were found, the student and professional groups (higher level of expertise) showing a slightly higher stability than the amateur group (lower level of expertise). The general coordination behavior as observed in the current study showed a high agreement with perceptual preferences reported in an earlier study to similar bowing patterns, implying that complex bowing trajectories for an important part emerge from auditory-motor interaction.

## Introduction

### Preludium

In violin and other bowed-string instrument performance, the primary function of bowing movements is to exert instantaneous control of the sound. In addition, bowing movements have to be planned ahead in order to anticipate future actions. Already in simple note sequences, this can lead to rather complex movement patterns, in which sound control, timing and anticipation are interwoven. Early observations by Hodgson obtained by means of cyclegraphy give a good impression of the wide variety of bowing movements that can be associated with excerpts from common musical repertoire [1].

**Figure 1:**
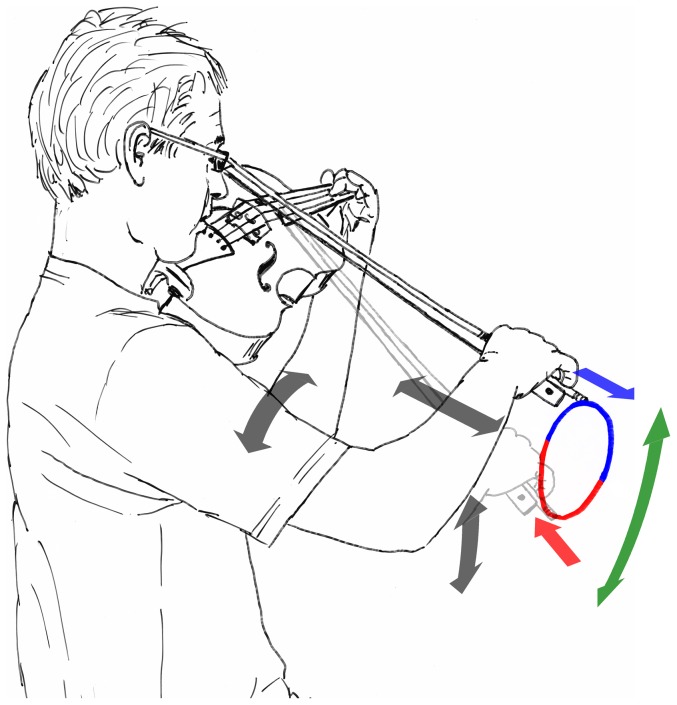
Movement components in fast repetitive bowing patterns. The grey arrows indicate the main degrees of freedom of the bowing arm when performing FRBPs. The trajectory of the bow (clockwise circular bowing pattern (CW) in this example) is indicated by the colored loop. The color indicates the bowing direction (radial movement component; blue = down bow, red = up bow). The green arrow shows the bow inclination (angular movement component), whose main function is selecting the string.

The focus of this paper is on a particular class of bowing movements, namely fast repetitive bowing patterns (FRBPs) involving simultaneous bow changes (i.e., reversal of the direction of the bowing movement perpendicular to the string) and string crossings (i.e., moving the bow from one string to another by pivoting it about the axis of the string(s)). The way in which such patterns are performed is demonstrated in Figure 1. The two movement components of the bow can be effectively described in a polar coordinate representation, where the to-and-fro movement (blue and red arrows) responsible for the production of sound is considered as the radial coordinate, and the pivoting movement (green arrow) responsible for string selection as the angular coordinate. The main bowing parameter associated with the former is *bow velocity*, which mainly controls the amplitude of the string vibration, and the latter corresponds to the *inclination* of the bow relative to the violin [2]. In the type of bowing patterns considered here, the radial component is predominantly produced by elbow flexion/extension, and the angular component by a combination of shoulder abduction/adduction and shoulder medial/lateral rotation. Thus, the respective movement components involve different groups of muscles, whose actions need to be coordinated to produce the desired behavior.

The resulting movement trajectories of the bow form fluent two-dimensional patterns, typically circular or figure-of-eight shaped. The relative timing of bow changes and string crossings, which is critical for an acceptably sounding performance, is inherent in the shape of the motion trajectory of the bow, and is achieved via a specific coordination of the two movement components. Preliminary observations by means of 3D motion capture revealed that in this type of bowing patterns, string crossings consistently preceded bow changes in all observed performances by several performers [3], [4]. This timing relation was achieved by a phase lead of bow inclination of about 10°–30° relative to bow velocity, both movement components being approximately sinusoidal as a function of time. Similar behavior was observed in more complex figure-of-eight patterns, in which bow velocity and bow inclination exhibit a 2∶1 frequency relationship.

Recently, it was shown in a perceptual study, in which participants could by means of a simple slider adjust the relative phase of bow velocity and bow inclination in a gesture-controlled virtual violin, that there was a clear preference for a similar phase relation between bow inclination and bow velocity [5]. This finding implies that the coordination behavior is tailored to the production of a desirable auditory outcome. This might not be surprising in itself since optimization of the produced sound is an essential aspect of musical instrument performance. It is, however, an interesting question how this specific behavior can be explained in terms of auditory- and other sensory-motor processes.

### Scientific context

Fast repetitive bowing patterns can be characterized as rhythmical movements performed at a relatively high movement frequency (typically 4–8 notes per second, corresponding to 2–4 complete cycles per second in case of circular patterns), which are subject to high spatiotemporal constraints. The timing relation between bow changes and string crossings is achieved through intralimb coordination, yielding a specific phase relation between two movement dimensions in the end effector. The primary source of feedback is the sound of the instrument, which has a direct relationship with the performer's actions in a complex and nonlinear manner [6]–[10], and reaches the ear of the performer in the form of a complex auditory stream. Finally, FRBPs represent a highly skilled type of action resulting from extensive training.

To our knowledge the study of this particular type of movement patterns is unprecedented in scientific literature on music performance and motor control. There are only few studies to bowing in string instruments, which focused on other aspects of motor behavior, such as intralimb coordination and control in the bowing arm during détaché bowing on a single string [11], [12], coordination between bowing action and finger actions in the left hand [13], [14], kinematics and kinetics of bowing arm movements with regard to overuse syndrome [15]–[17], model-based analysis of effort in a variety of typical bowing techniques [18], motor learning and group differences between novices and advanced performers in basic bowing skills [19], [20], and bow-control strategies in sustained notes [10].

In terms of motor control, coordination in FRBPs can be compared to intralimb, or intersegmental, coordination in circle drawing [21], [22], handwriting [23], [24], and other types of arm movements [25]. Coordination in rhythmic movement behavior has been successfully modeled in terms of nonlinear dynamical system theory [26]–[28]. Studies on coordination have mainly been focused on phase relations in rhythmic bimanual patterns. Kelso et *al*. revealed a clear preference for in-phase and anti-phase modes of behavior, as well as a non-linear transition from anti-phase to in-phase behavior when increasing the movement frequency, which was attributed to a preference for co-activation of homologous muscles [26], [29]. More recent studies have shown the importance of perceptual information in the formation of stable coordination patterns [30], [31]. In particular, it has been shown that by providing appropriate visual feedback, bimanual coordination patterns could be sustained that otherwise would be difficult, if not impossible, to achieve [32]–[35]. These results suggest that coordinated behavior emerges as the result of a coalescence of constraints associated with sensory input and motor output, which need to be modeled in an integral approach [31], [36], [37].

From a neuroscientific perspective, musical performance presents an interesting case for the study of auditory- and sensory-motor interaction [38], [39]. Sensory-motor processes can be roughly categorized into two basic types of interaction. The first type concerns adaptation of motor behavior in reaction to sounds from the environment, here considered as open-loop interaction. This type of sensory-motor interaction has received much research attention, for example in sensory-motor synchronization experiments (see [40], [41] for a comprehensive review), and in clinical applications [42]. The second type concerns feedback from self-generated actions, here considered as closed-loop interaction. Violin bowing can be considered as a typical example of this second type of interaction. Closed-loop interaction has been extensively studied in the perception and production of speech [43]. In music performance it has been shown that sensory feedback is related to the precision of timing of musical events [44]–[46]. Other studies have demonstrated that delay of auditory feedback can lead to disruption of performance, in particular with regard to timing [47], and that it can influence kinematic features during a rhythm production task [48]. An emerging area of research is concerned with the use of sonification of movement as a means of feedback for learning and optimization of complex skills, which has interesting potential for applications in sports and rehabilitation [49], [50].

It should be noted that purely open-loop interactions as defined above do not exist in isolation in humans and other living creatures under normal conditions, given that any action is accompanied by some kind of self-generated sensory feedback. Moreover, many activities involve a mutual interaction with other entities and/or the external environment, which requires a combination of open- and closed-loop interaction. Such interactions have recently been studied empirically in musical ensemble performance [51]–[56], adaptive sensory-motor synchronization [57], [58], and other types of joint behavior [59], [60].

### Aims

The experiment reported in this paper forms part of a larger study of perception-action coupling in the coordination of complex bowing patterns. The focus of the current study is on motor-behavior aspects of coordination in fast repetitive bowing patterns. By including a relatively large number of performers for this type of study, we aimed to gain insight in common aspects of behavior, as well as inter-individual variability. The performers were asked to play different patterns in a variety of conditions (tempi, dynamic levels and string combinations) in order to assess the robustness of coordination behavior, as well as the ability of performers to adapt to different performance constraints. Moreover, the influence of skill level on coordination behavior and stability was investigated by including three groups of performers (amateurs, students, professionals). The analysis was restricted to movements of the bow, which is here considered as the end effector.

An important intention of this study is to explain the observed motor behavior in terms of its functional context, namely sound production on the violin. This requires an interdisciplinary approach making a connection between the realms of music acoustics and motor control.

### Coordination model

Before proceeding to the results, it is crucial to have a basic understanding of the acoustical constraints associated with note transitions in FRBPs.

**Figure 2:**
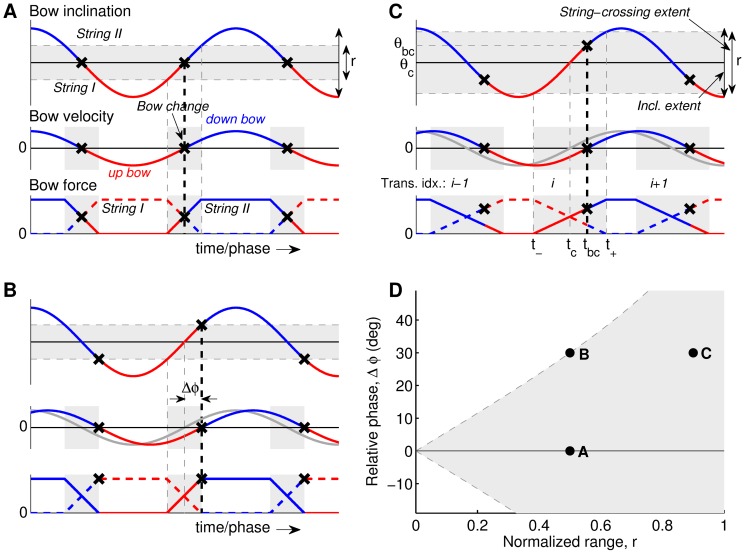
Simplified coordination model. Panels A–C show the relation between bow inclination, bow velocity and bow force vs. time in three different situations. The grey areas indicate the angular area or the time intervals in which the bow traverses from one string to another (string crossings). The bowing direction is indicated by the color of the curves (blue = down bow, red = up bow), and bow changes are marked with an **x**. The bow-force subpanels show the bow force exerted on both strings, which are distinguished by the line style (solid and dashed, respectively). The respective panels show: (A) Basis case with zero phase difference (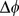 = 0, 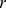 = 0.5), (B) a phase lead of bow inclination with respect to bow velocity (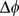 = 30°) at the same value of 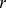 (in the figure shown as a phase lag of bow velocity, relative to the unshifted grey line), and (C) same phase lead at a large normalized string-crossing range (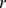 = 0.9). Panel C includes annotations indicating the features extracted for analysis (see “Materials and Methods: Feature extraction”). Panel D shows the situations in panels A–C as points in the 2D coordination space 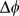 vs. 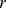. The grey area represents the combinations of 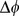 and 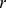 at which bow changes take place within the string-crossing area.

Coordination in FRBPs can be described by a simplified model proposed in [5]. The model provides useful insight into the relation between the bowing movements and the conditions for vibration of two adjacent strings during note transitions. Three situations are sketched in Figure 2. The panels A, B and C in Figure 2 contain three sub-panels showing bow inclination, bow velocity, and normal force exerted by the bow on the two individual strings. Both bow inclination and bow velocity are modeled as sines as a function of time, and the total bow force (distributed across the strings in contact with the bow) is constant. An essential aspect of the model is that it takes into account the non-zero angular range of string crossings due to the compliance of the bow hair and the strings [61], as opposed to instantaneous string crossings. This is indicated in the inclination sub-panel by the grey area in which the bow is in contact with both adjacent strings. The grey areas in the two lower sub-panels indicate the corresponding time intervals during which bow force is transferred from one string to the other in a cross-fade-like manner.

The two main coordination parameters in the model are the relative phase 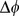 between bow inclination and bow velocity (defined positive for a phase lead of bow inclination), and the width of the string-crossing range 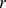 normalized with respect to peak-to-peak inclination extent. Figure 2A shows a baseline situation in which 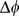 is zero. The influence of relative phase becomes clear in Figure 2B, where it can be seen that it leads to a shift of the moments of the bow changes relative to the string crossings (bow inclination signal). In the sketched situation, the bow changes take place at the moment that the string crossing is completed, and bow force is fully transferred to the “new” string. Finally, Figure 2C shows the influence of 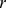, which in the sketched situation is increased while 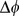 remains the same. Due to the increase of 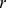, the bow change now takes place well within the string crossing again, leading to altered conditions of the transition.

Coordination strategies can be effectively displayed in a two-dimensional coordination space of 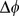 vs. 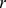, as shown in Figure 2D. The grey area represents the area in which bow changes take place within the string-crossing range, which is delineated by the curves 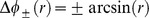 (see [5] for a more detailed derivation). The points corresponding to the situations sketched in panels A, B and C are indicated in the plot.

It has been argued in [5] that there is no obvious solution to the problem of coordinating bow changes and string crossings. The reason for this is that, besides the main attack (i.e., the note onset on the “new” string at the bow change), the transition includes additional events that influence its perceptual quality. We can distinguish two types of *false attacks*, the first one (type I) associated with the “new” string at the moment that the bow enters the string crossing range (assuming that the bow change takes place after that), and the second one (type II) associated with the new string at the moment of the bow change (provided that it takes place within the string crossing range). Furthermore, there might be remaining vibrations in the “old” string dependent on how effectively it has been stopped at the end of the transition. The prominence of these additional events, as well as the quality of the main attack, depend on the combination of the two coordination parameters. As a result, the optimization of note transitions involves a trade-off based on the perception of these features, which could be confirmed by an acoustical analysis of the stimuli used in the above-mentioned perceptual experiment [62].

The coordination strategies observed in performance will be interpreted in terms of the simplified model. However, even though this model provides a good first order description of FRBPs, we might expect some important departures from it in actual performance. First, in the model it is assumed that the bow inclination movement is centered at the middle of the string crossing. However, since bow inclination has no absolute zero, it might be offset in performance, leading to asymmetry between transitions. Second, bow force might fluctuate in performance, which in turn leads to fluctuations of the width of the string-crossing range [61]. Finally, bow inclination and bow velocity might not be perfectly sinusoidal, which might lead to within-period fluctuations of relative phase. In effect, these departures provide additional degrees of freedom to the performer, which need to be considered for a complete and correct understanding.

### Expectations

In the following, we refer to coordination strategies as the combination of coordination parameters (relative phase, normalized range and inclination offset) used by the performers to achieve their preferred sound. In accordance with other coordination studies (e.g., [22], [25], [32]–[35], [63]), we consider the variability of relative phase as an indicator of coordination stability. In addition, we consider the other coordination parameters (normalized range and inclination offset) as performance variables to be stabilized, given their potential influence on the sound quality in these particular bowing patterns.

Based on earlier observations and results from a perceptual study of coordination in FRBPs described above, we hypothesize a consistent phase lead of bow inclination relative to bow velocity across participants and conditions. Furthermore, we expect that the coordination strategies might depend on the factors controlled in this study in a partly predictable way. However, it should be noted that the coordination behavior might involve complex interactions with other bowing parameters, such as bow velocity, bow acceleration, bow force and bow-bridge distance. Whenever necessary, the possible relations with these parameters will be discussed.

Concerning *level of expertise*, we might expect that more skilled performers show a larger inter-individual agreement concerning their coordination strategies within a given condition compared to less skilled performers, approaching an optimal strategy, provided that it exists. Furthermore, we expect that they are better able to adapt to a wide range of performance conditions (e.g., bowing pattern, tempo, dynamic level), and that they show a higher intra-individual stability.

The experiment involves three patterns with different *levels of difficulty*. The clockwise circular pattern (CW) is most basic and already occurs in repertoire after about one year of lessons. The anti-clockwise pattern (ACW) is more difficult to perform, especially at high tempo, probably due to biomechanical constraints. Performers usually try to avoid it by simply inverting the bowing direction. (The issue of a preferential bowing direction with regard to string crossings is discussed in Szende and Nemessuri [64], where it is explained that in preferred arpeggios both parts of the arm move towards or away from rest position, whereas in reversed arpeggios the parts of the arm are forced to move in opposite directions in relation to the rest position. This notion is consistent with observations by Li et *al*. [65] that isodirectional intralimb coordination patterns were more stable than non-isodirectional ones. However, it should be noted that at high movement frequencies as in the current study, the influence of interaction torques forms a complicating factor, necessitating a more advanced dynamic analysis.) Finally, the figure-of-eight pattern (Fo8) is considerably more complex as it involves a 2∶1 frequency relationship between the radial and the angular movement components, and it requires deliberate practice even by more skilled players before it is mastered. It is therefore expected that 1) the stability of CW is highest, followed by ACW and Fo8, and 2) the more difficult the pattern, the more difficult it is to achieve an acceptable coordination strategy, especially at higher tempi.

Performance tempo is associated with the *frequency* of the movements. At higher tempi the movement amplitudes might become more constrained, which might lead to larger values of 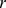 (mainly due to a decrease of inclination amplitude). There are no obvious constraints with regard to 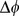, which therefore is expected to be rather independent of tempo. However, at lower tempi it could be expected that the movements gradually change from a continuous (quasi-sinusoidal) character to a more intermittent character, which might lead to larger within-period fluctuations of relative phase. The effect of tempo on stability is difficult to predict. On the one hand, coordination control might become more difficult at higher tempi, which would lead to increased variability. On the other hand, at lower tempi it might be possible that a transition of dynamic behavior is approached according to an earlier report [66], which might in its turn give rise to increased instability (critical fluctuations).

Dynamic level is associated with the *amplitude* of the movements. An increase in dynamic level is usually achieved by an increase in bow velocity and bow force [7], [10]. The latter will lead to an increased width of the string-crossing area, which therefore requires the amplitude of the inclination movement to increase when 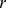 is to be kept constant. However, the inclination amplitude might be constrained at high dynamic level due to the vicinity of next-neighbor strings, which are not supposed to be involved. In that case, it might be expected that 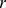 takes higher values at high dynamic levels. There are no obvious constraints with regard to 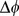, which therefore is expected to be independent of dynamic level. Finally, it might be expected that the smaller and less firm movements associated with performance at low dynamic level lead to a higher variability.

Lastly, the physical properties of the strings might also have an influence on the coordination behavior. Lower strings have a higher characteristic impedance, and therefore require a different combination of bow acceleration (lower) and bow force (higher) for a clean attack [8]. At lower strings it might therefore be of higher importance that bow force is fully transferred to the new string at the moment of the bow change, which requires that the bow change takes place when the string crossing is completed (i.e., 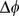 should be large enough in relation with 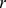). The coordination strategy might therefore depend on the used string combination.

## Materials and Methods

### Ethics statement

The study was approved by the local ethics committee (Hanover Medical School, No. 1253–2011) and conducted according to the declaration of Helsinki. All participants provided their written informed consent to participate in the study. Three of the participants were at minor age (one at the age of 16, and two at the age of 17) at the time of the experiment. For these participants additional verbal consent was obtained from their parents, which was documented in the participant administration files. Written consent from the parents was not obtained since they could not be present during the experiment. It was therefore agreed that written consent could be obtained from the participants themselves. This consent procedure was approved by the local ethics committee.

### Participants

Twenty-two violin players participated in the experiment (age (mean, stdev) 28.0±9.8; 15 female, 7 male; 20 right-handed, 1 left-handed, 1 unknown). The participants were categorized into three groups with different level of expertise, namely amateurs (lowest level of expertise), students (intermediate level of expertise), and professionals (highest level of expertise). The groups were composed as follows: amateurs (8 participants: age 26.3±6.4; 7 females, 1 male, all right handed), students (8 participants: age 21.5±3.8; 5 females, 3 males; 7 right-handed, 1 left-handed), and professionals (6 participants: age 38.8±10.4; 3 female, 3 male; 5 right-handed, 1 unknown). All participants received a 30 EUR fee for their cooperation.

The following recruitment criteria were employed. All participants (including the amateurs) had to play at a sufficiently advanced level to be able to perform pieces like the Preludium of the third Partita for solo violin by J.S. Bach in order to make sure that they could play the bowing patterns asked in the experiment. The advanced amateur players were recruited from amateur and youth orchestras in Lower Saxony. Amateur players who previously had been enrolled in music studies with violin as a principal instrumental study subject were excluded. At the time of the experiment three of the amateur participants (AM3, AM5 and AM7) were or had been enrolled in music education studies. Inclusion criteria for the student group were that the participants were currently enrolled as a bachelor or master student in music performance with violin as principal instrumental study subject. All participating students were recruited from the Hanover University of Music, Drama, and Media, and they studied with in total three different professors. For the professional group, elite violinists were recruited with an active career as a soloist, chamber musician, or section leader in a professional orchestra. The elite participants came from all over Germany.

Additional criteria for assessment of skill level were obtained by means of a debriefing questionnaire, which the participants were asked to complete after the experimental session. The questionnaire consisted of four parts, including 1) a shortened version of the Edinburgh Handedness Inventory [67], [68] (see: http://bit.ly/MTpnRR), 2) the Ollen Musical Sophistication Index questionnaire [69], 3) an estimation of the number of cumulative practicing hours as a function of age [70], and 4) a question with regard to current performance experience. With regard to cumulative practice hours (item 3), the question was formulated as follows: “Please give a rough indication of how much time you spent on practicing the violin in different periods of your life. You are free to make your own subdivision that makes most sense for you, for example per teacher or per period of five years.” The participants responded in a tabular format, specifying the periods in terms of age and the associated amount of practice in hours per day. This also yielded an estimate of the current amount of daily practice, which might be an interesting indicator with regard to fitness of motor skills [71].

The expertise measures were not used in the analyses. The participant information is available as supporting information (Dataset S2, see Dataset S1 for a detailed description of the dataset).

### Design and Procedure

The total duration of the experiment was about 2.5–3 hours, including preparations, recordings and the filling out of the debriefing questionnaire. The participants were asked to wear a motion-capture suit. Motion-capture markers were applied using a custom full-body configuration. All participants used the same master-quality violin and bow, which were prepared in advance for the measurements (see details below). The participants were standing during recording. Before the start of the recordings, the participants were given time to familiarize with the experimental situation, making sure that they could play comfortably. The participants were informed that they could take a break at any moment during the experiment, and they were encouraged to do so in between the different parts of the experimental session. In case a metronome was used (in the tempo conditions), the clicks were presented by an open earplug (Sennheiser OMX 180) in the right ear only.

The recording sessions consisted of six parts, forming a balanced mix of controlled conditions and musical fragments. The main part of the experiment was focused on fast-repetitive bowing patterns in a musical context (Preludium of the third Partita for solo violin by J.S. Bach), as well in elementary note patterns at a variety of dynamic levels and tempi (controlled FRBP conditions). An additional part was focused on the production of inaudible bow changes (not analyzed in the current paper), and at the end of the experiment participants were asked to perform a musical piece of their own choice (for exploratory purposes).

**Figure 3:**
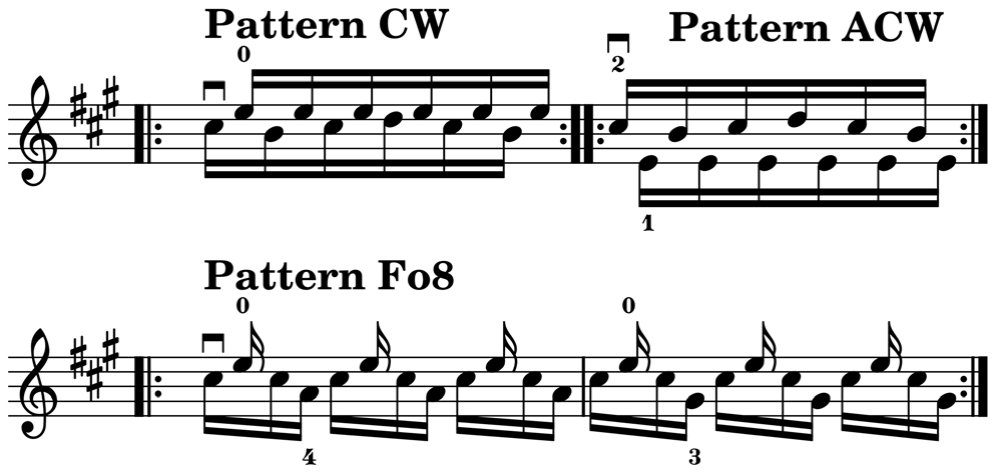
Note patterns. Musical notation of the three bowing patterns (CW = clockwise, ACW = anti-clockwise, Fo8 = figure-of-eight) used in the experimental conditions.

The three note patterns used for the controlled FRBP conditions are shown in Figure 3. The circular clockwise (CW) and anti-clockwise (ACW) patterns are characterized by a 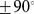 relative phase in the spatial domain (not including 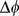). The figure-of-eight (Fo8) pattern is characterized by a 2∶1 frequency relation and a relative phase of zero, yielding a figure-of-eight shape in the spatial domain.

In order to make the recording sessions as efficient as possible, the controlled FRBP conditions were organized in a total of six recording blocks: two dynamic-level blocks and four tempo blocks. Each recording block consisted of three to four conditions, which were played three times each, yielding a total of 9–12 sequences per block. Each sequence consisted of six seamlessly repeated bars (see note patterns in Figure 3), yielding a total of 72 notes per sequence. The order of the sequences within each recording block was randomized. During the tempo conditions the participants synchronized to a metronome throughout the performance in order to avoid possible tempo drift.

The recording sessions were structured as follows:

1. Bach Preludium: 2 fragments containing CW and Fo8 patterns, free choice of tempo (optional, about 5 min.; not considered in this paper)
2. Two dynamic-level blocks: forte and piano (random order); consisting of CW and Fo8 patterns in two transpositions (as written in Figure 3, and one fifth lower, i.e., the same fingering one string lower); tempo instructions: between 92–112 bpm, without metronome; each condition was repeated three times (12 sequences per block, total duration about 10 min.)
3. Bow changes (not considered in this paper, about 15 min.)
4. Four tempo blocks: 58, 72, 92 and 112 bpm (random order), with metronome; consisting of CW, ACW and Fo8 patterns, each repeated three times (9 sequences per block); in addition a “slow” block was recorded in which each pattern was played at 29 bpm (total duration about 15 min.)
5. Bach Preludium (optional, not considered in this paper)
6. Musical piece of participant's own choice (optional, not considered in this paper)

In the current analyses only the dynamic level (item 2) and the tempo conditions (item 4) were considered. An overview of these conditions is presented in Table 1. The note rates at the indicated tempi (58, 72, 92, and 112 bpm) are 3.9, 4.8, 6.1, and 7.5 notes/s, respectively, corresponding to movement frequencies of 1.95, 2.4, 3.05, and 3.75 Hz for the circular patterns. The total number of controlled conditions was 20 (not including the “slow” conditions). The effective playing duration of these conditions was about 12:30 minutes per participant, yielding a total of more than 4:30 hours of playing for all 22 participants. A video example showing visualizations of the three bowing patterns is provided as supporting information (Video S1–S3).

**Table 1:** Condition overview.

block	tempo	dyn. level	pattern	str. combi	N
Dynamic level	92–112^1^	forte	CW, Fo8	E-A/A-D, E-A-D/A-D-G^2^	4
Dynamic level	92–112^1^	piano	CW, Fo8	E-A/A-D, E-A-D/A-D-G^2^	4
Tempo	58	forte	CW, ACW, Fo8	E-A, A-D, E-A-D^3^	3
Tempo	72	forte	CW, ACW, Fo8	E-A, A-D, E-A-D^3^	3
Tempo	92	forte	CW, ACW, Fo8	E-A, A-D, E-A-D^3^	3
Tempo	112	forte	CW, ACW, Fo8	E-A, A-D, E-A-D^3^	3
Slow	29	forte	CW, ACW, Fo8	E-A, A-D, E-A-D^3^	3
Bach Preludio	92–112^1^	forte/piano	CW, Fo8	E-A/A-D, E-A-D/A-D-G^2^	8

1 Tempo indication, no metronome or cue.

2 Two string combinations per respective pattern.

3 Fixed string combination per respective pattern.

Overview of experimental conditions or tasks: tempo (in bpm), dynamic level (forte = loud, piano = soft), note/bowing pattern (CW = clockwise, ACW = anticlockwise, Fo8 = figure-of-eight), and string combination (E, A, D, G from high to low pitch). The left-most column (N) indicates the number of conditions included in the respective blocks.

### Equipment

Motion-capture data were recorded using a Qualisys Oqus 3+ passive optical motion-capture system consisting of seven cameras in a circular configuration around the participant. The cameras were positioned at a height of about 2.8 m, and the radius was about 2.5–3 m. The motion-capture sample rate was 240 Hz. Audio (48 kHz, 16 bit) and video (30 Hz) were synchronously recorded with the motion capture data, and were mainly used for control purposes.

A custom marker configuration, especially suitable for the analysis of upper-body and arm movements, was used for measuring full-body motion. (An analysis of the body-motion data falls outside the scope of the current paper and will be presented elsewhere.) The violin and the bow were equipped with 5 markers each, using a similar marker configuration as described in [2], allowing for accurate 6DOF tracking. In addition, the bow was equipped with a custom-made sensor for measurement of bow force [72] and a miniature 3D accelerometer (ST, type LIS344ALH, linear range ±6 g). The sample rate of sensor data was 1.2 kHz (5 times the motion-capture sample rate). The total mass added to the bow was about 14 g, mainly concentrated in the lower part of the bow (closest to the hand of the player). All participants reported that they were able to perform normally. One participant (STUD2) made a critical remark about the added mass.

### Data analysis

#### Bowing data

Bowing data (bow velocity, bow force, bow-bridge distance, bow inclination, etc.) were obtained from the 6DOF data of the bow and the violin and the sensor signals as described by Schoonderwaldt and Demoucron [2], [72]. This involved a series of calibrations at the start of each experimental session in order to obtain the string positions, the position of the bow-hair ribbon, the angles between the strings (in terms of bow inclination), and the position-dependent sensitivity of the bow-force sensor. Geometrical landmarks on the bow and the violin were obtained using a custom-made digitizing probe with an estimated accuracy of about 0.3 mm. Inverse kinematics calculations were done using the algorithm described by Veldpaus et *al*. [73]. The width of the string-crossing areas in terms of bow inclination was calculated as a function of bow force and bow-bridge distance using the method described in [61]. This required knowledge of the compliance of the strings (based on manufacturer specifications of string tension) and the bow-hair ribbon (estimated from the bow-force-sensor calibration measurement). In addition, a correction was applied for displacement of the strings when stopped by a finger of the left hand, based on a (pre-calibrated) geometrical model of the fingerboard [61]. All calculations were performed in Matlab.

Since motion-capture data are inevitably noisy, the following filtering procedures were adopted. 1) Due to partial occlusions by the bow, there was a regular occurrence of large jumps (up to 1 cm) in some of the markers on the violin. These jumps caused noticeable orientation artifacts, which were propagated in the estimation of bow inclination. The artifacts could be effectively removed by applying a rather strict low-pass filter (4*^th^*-order Butterworth, cut-off 4–6 Hz) to the violin marker positions prior to the inverse kinematics calculations. Application of such a strict filter was judged viable for the purposes of this study since the movements of the violin were much smaller than those of the bow. A graphical check was routinely applied to make sure that there were no noteworthy filter artifacts introduced by this procedure. 2) Bow velocity was low-pass filtered (2*^nd^*-order Butterworth, cut-off 30 Hz) in order to reduce high-frequency noise due to differentiation. 3) The estimated string-crossing width was low-pass filtered (2*^nd^*-order Butterworth, cut-off 48 Hz) in order to reduce the influence of noise from the bow force sensor. All Butterworth filters were applied back-and-forth in order to avoid phase shifts.

#### Selection procedure

A semi-automatic procedure was used for selecting the performance fragments from the recording blocks. The start and the end of the fragments were marked by mouse clicks. The audio of the selected part was then automatically played, allowing the experimenter to exclude parts containing obvious performance errors (erratic notes), or synchronization errors in case a metronome was used, which could have disrupted the ongoing coordination behavior. There were only infrequent occurrences of such errors. The first and the last beat of the selections (four notes each) were automatically discarded since we were only interested in stable coordination behavior. During the selection procedure, meta-data were automatically assigned (under supervision of the experimenter) based on the particular protocols of the experimental sessions, facilitating further automatic processing of the data.

#### Calculation of relative phase

The calculation of the phase difference between bow velocity and bow inclination (relative phase, 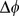) was based on the Hilbert transform, which provides a robust estimate of continuous phase of quasi-sinusoidal signals without frequency artifacts [74]. First, the continuous phase signals of bow velocity and the first derivative of bow inclination (angular velocity, low-pass filtered, cut-off 30 Hz) were estimated from their respective Hilbert transforms. The advantage of using velocity signals is that they are naturally zero centered, avoiding the use of (arbitrary) amplitude centering corrections and possible artifacts due to baseline fluctuations. The continuous relative phase was then obtained by subtraction of the two phase signals. For the figure-of-eight (Fo8) pattern, the continuous phase signal of bow velocity was divided by a factor two in order to compensate for the 2∶1 frequency relation of bow velocity and bow inclination. (Initially, it was decided to calculate the phase relative to the inclination signal, so that it relates to a full cycle of the bowing pattern. For this reason the Hilbert phase of bow velocity was divided by a factor 2 before subtraction. However, for the sake of comparability with circular patterns, it is more correct to take bow velocity as a reference. For this reason the relative phase of the Fo8 pattern was eventually multiplied by a factor two for the statistical comparisons.) For each pattern a constant phase offset (closest integer multiple of 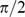) was subtracted in order to obtain comparable measures of the phase difference relative to zero (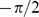, 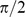, and 0 for patterns CW, ACW and Fo8, respectively).

#### Feature extraction

For the statistical comparisons, discrete features were extracted from the continuous bowing data. Bow changes were detected by zero crossings in the bow-velocity signal. For each bow change an additional set of features was extracted. An overview of low-level features associated with a transition is shown in Figure 2C. These low-level features were subsequently used for calculation of higher-level features (coordination parameters). Sub-sample estimates of the moment of the bow change (*t*_bc_) and other time points, as well as their associated features, were obtained by linear interpolation. All bow changes were indexed, allowing to distinguish between contextual features, such as bow-change direction (from down to up bow and vice versa), string combination, metrical position (bar, beat, sub-beat), etc.

The high-level features used in the statistical comparisons are defined as follows.

- Relative phase (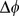): relative phase value at the moment of the bow change *t*_bc_
- Normalized range (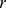): width of the string-crossing range at *t*_c_ divided by the peak-to-peak inclination extent (peak values before and after the bow change)
- Inclination offset (*θ*_offset_): angular offset relative to the center of the string crossing, calculated as the average of the inclination values at the current and the preceding bow change,

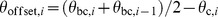



It should be noted that the normalized range 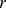 is not defined for pattern Fo8 since the peak-to-peak extent of inclination has a different meaning in the context of the two string crossings involved. Furthermore, the estimation of relative phase based on the respective Hilbert phases of bow velocity and angular velocity can only be well interpreted as long as both signals are quasi-sinusoidal as a function of time. The latter criterion might be violated, especially at lower tempi. In particular, the inclination movement in pattern Fo8, which by definition is twice as slow, resembled more a triangular shape (i.e., linearly changing inclination rounded at the inversions), especially at lower tempi, and since the estimation of the phase angle is based on its derivative, this resulted in strong local fluctuations in the relative phase signal. These problems can be circumvented by taking equivalent time-domain estimates of relative phase and normalized range, which are defined as follows.

- Relative phase (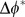): time difference between bow change and moment of intersection with the string-crossing center, normalized with respect to cycle duration,

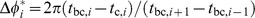

- Normalized range (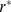): based on the duration of the transition, normalized with respect to cycle duration, assuming a sinusoidal model,

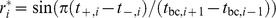



Comparison of the regular and the alternative (time-domain) estimates averaged per participant and condition (for details, see feature statistics below) showed that there was a large general agreement, with some noteworthy exceptions. In the two slowest tempo conditions (58 and 72 bpm) in pattern Fo8, the Hilbert-based estimate of relative phase was up to a factor two larger, which could be attributed to the triangular shape of the bow inclination signal. In these conditions the time-domain estimate was considered more accurate. When leaving out these two conditions, the correlation coefficient between the two estimates of relative phase was 0.95 (slope 0.85, alternative vs. regular), increasing to 0.98 (slope 0.93) when completely leaving out pattern Fo8. With regard to normalized range, the correlation coefficient between the two estimates was 0.99 (slope 0.94), not including pattern Fo8, for which the regular estimate 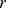 is not defined.

Comparison of the standard deviations of the estimates (per participant and condition) showed a good agreement for normalized range with a correlation coefficient of 0.91 (slope 0.69). For relative phase the correlations between the standard deviations of the estimates were much lower, and showed a large discrepancy between pattern Fo8 and the two circular patterns (CW and ACW). For the combined circular patterns the correlation coefficient was 0.71 (slope 1.50), and for the Fo8 pattern the correlation coefficient was 0.64 (slope 0.61). For the circular patterns the standard deviations of the time-domain estimate were larger. A possible explanation is that the time-domain estimate contains an additional source of error, namely the fluctuation of the center of the inclination signal, which is reflected in the estimation of 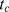. This becomes more influential at higher tempi since the normalization then involves division by a smaller time interval, which leads to inflation of the variance. For pattern Fo8, the larger standard deviation of the Hilbert-based estimate could be partly attributed to the strong local fluctuations in the relative phase signal due to the non-sinusoidal character of the bow inclination signal.

Summarizing, the regular and the alternative estimates of the coordination parameters contain different strengths and weaknesses, which need to be taken into account when doing statistical comparisons of coordination strategies and stability. Concerning normalized range, the two estimates were practically identical, both with regard to the mean and the standard deviation. Concerning relative phase, there was a good agreement between the means with exception of pattern Fo8; however, the standard deviations showed important deviations, which could be related to particular strengths and weaknesses of the respective measures.

The dataset of means and standard deviations of features per participant and condition is available as supporting information (Dataset S3, see Dataset S1 for a detailed description of the dataset).

#### Feature statistics

For statistical comparisons, means and standard deviations of the features per participant and condition were collected in a data table. This was done in two steps. First, the means and standard deviations of the features were calculated per participant, condition and transition (two transitions for the circular patterns and four transitions for the Fo8 pattern). Then, the means and standard deviations were averaged across transitions.

Statistical analysis was performed in R [75]. Mixed between- and within-subject ANOVAs were done using the package “ez” [76]. As the level-of-expertise groups were unbalanced, type II sums of squares were used [77]. If relevant, sphericity-corrected p-values are reported for within-subject factors with more than 2 levels. Generalized eta squared values (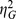) are reported as effect sizes; effects are considered small from 0.02 to 0.13, medium from 0.13 to 0.26, and large for 0.26 and above [78]. For multiple comparisons of means, Tukey contrasts with Holm-adjusted p-values were used.

## Results

The Results section is structured as follows. First, we present the overall distributions of the main coordination parameters, followed by a graphical overview of individual coordination strategies in the basic conditions. Second, we present an analysis of circular clockwise bowing patterns in the dynamic-level conditions. Third, we present an analysis of the tempo conditions, including the three bowing patterns (clockwise, anti-clockwise, and figure-of-eight). An interpretation of the results in terms of influences of the experimental factors on coordination strategy and stability is presented in the Discussion section.

### Distribution of main coordination parameters

**Figure 4:**
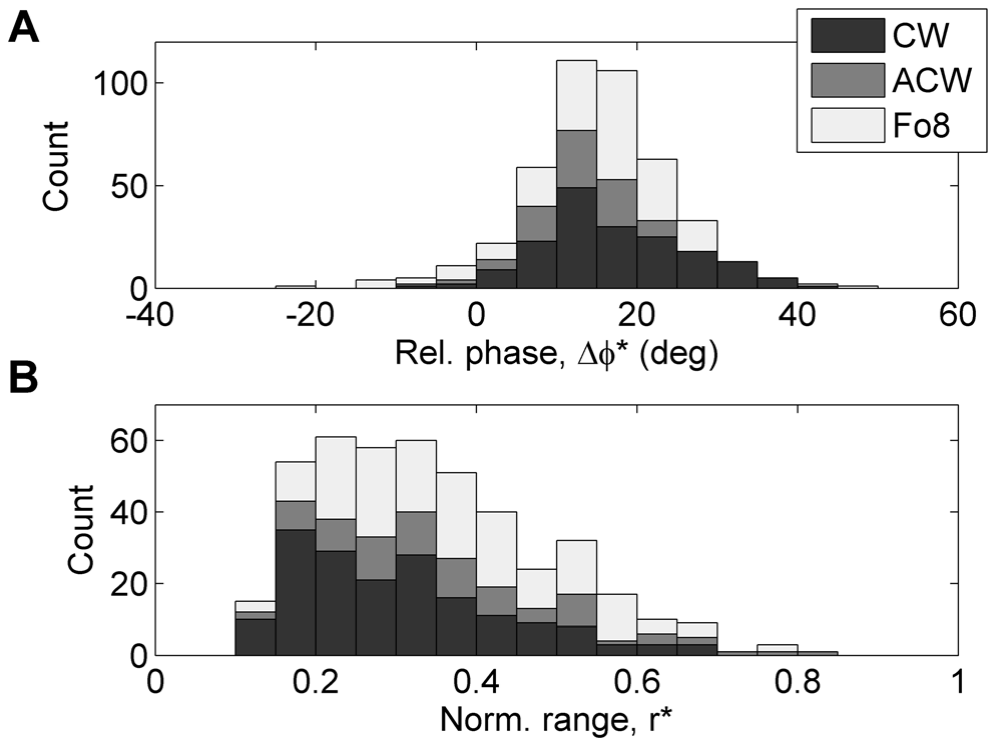
Distributions of main coordination parameters across all dynamic level and tempo conditions by all participants (N = 436). The panels show (A) relative phase (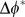), and (B) normalized range (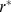). The three bowing patterns are distinguished by their grey shade (see legend).

The distributions of the main coordination parameters 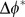 and 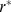 across all conditions (dynamic level and tempo) by all participants are shown in Figure 4. In this overview the time-domain estimates of the coordination parameters were selected so that pattern Fo8 could be included in the overview. Panel A clearly shows that the relative phase was larger than zero in a large majority of the performances. The median was 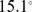, and 80% of the values fell into the range of 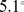 to 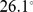. A one-tailed t-test of relative phase averaged per participant indicated that the true mean (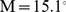) was significantly greater than zero [t(21) = 14.7, p<0.001]. There were only few occurrences of negative values of relative phase, which could be mainly attributed to pattern Fo8. The other occurrences were mainly associated with one of the student participants, who featured exceptionally low values of relative phase in some of the conditions. The overall median value of 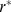 (panel B) was 0.32, and 80% of the values fell into the range of 0.18 to 0.54. The distributions of relative phase and normalized range showed remarkable similarities to those found in a perceptual study of similar bowing patterns [5].

### Coordination strategies in basic conditions

**Figure 5:**
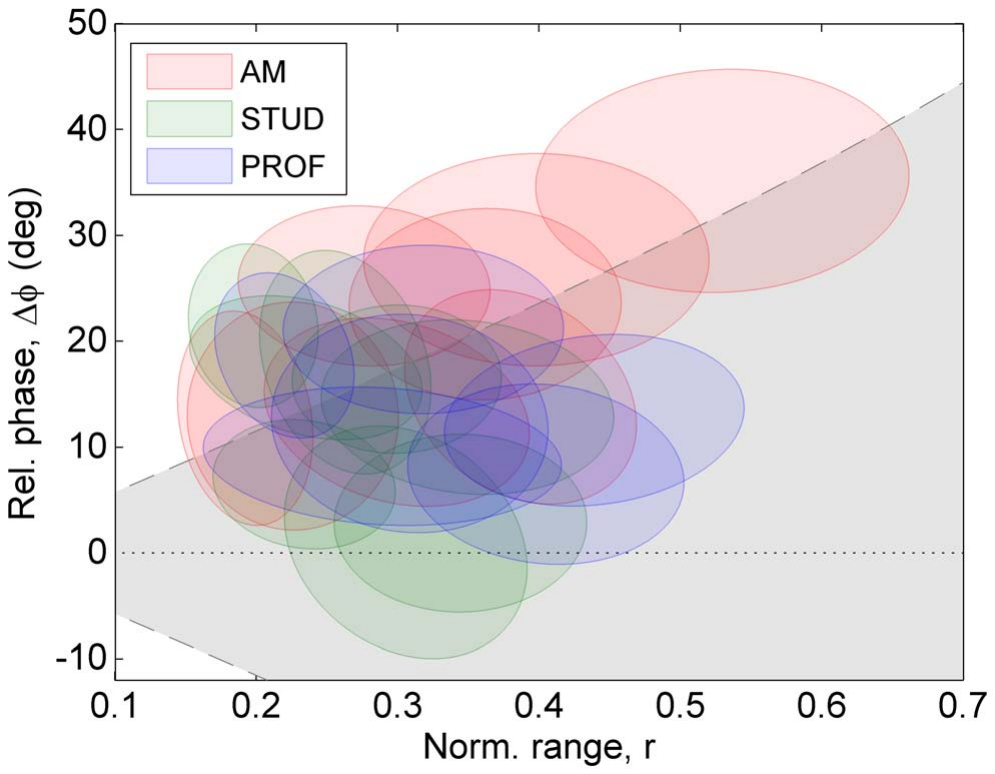
Overview of the individual coordination strategies of all participants in the basic conditions (clockwise circular pattern, performed forte at moderate to fast tempo). Each individual participant is represented by an ellipse showing the average (center) and one multivariate standard deviation (i.e., 68% of the data falls within the ellipse). Skill level is indicated by color (see legend). The grey area represents the combinations of 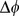 and 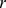 at which bow changes take place within the string-crossing area.

Figure 5 shows an overview of individual coordination strategies, representing an average of three basic (fast-forte) conditions [pattern CW on E-A string combination: tempo 92, 112 bpm (tempo block), and forte (dynamic-level block)]. Based on this figure the following observations can be made. First, most data were clustered around a relative phase value between 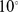 and 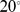 and a normalized range of about 0.3. Furthermore, the individual ellipses showed a scattered distribution with only a limited amount of overlap, which indicates that the inter-individual agreement was small, even among the professional participants, whereas the relatively small sizes of the ellipses demonstrate a high within-individual consistency. This could be confirmed for the respective dimensions by t-tests (one-tailed), comparing the individual standard deviations to the standard deviation of the collective mean [for 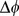: true mean of the individual standard deviations (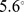) is less than the standard deviation of the collective mean (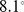), t(21) = −12.3, p<0.001; for 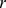: true mean (0.056) is less than 0.084, t(21) = −8.5, p<0.001]. Finally, only minor distinctions between the groups of different level of expertise (indicated by color) can be observed; in the amateur group (red) 2–3 participants showed a combination of high 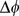 and 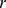, whereas in the student group (green) 2–3 participants showed rather low values of 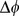. However, a one-way MANOVA revealed no significant effect of group on the used combination of 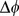 and 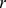.

### Dynamic level, string combination and expertise

**Figure 6:**
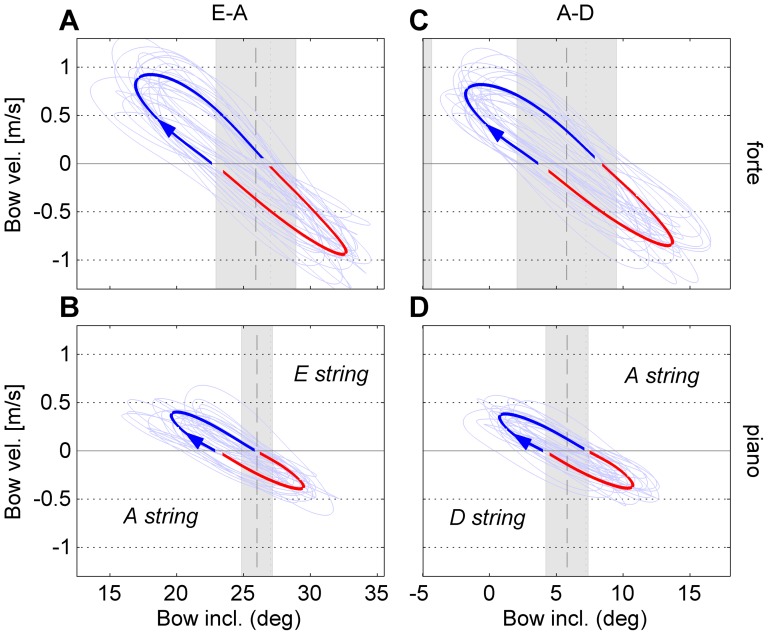
Coordination patterns of the four clockwise-pattern conditions in the dynamic-level block. The plots represent the grand average across all participants of bow velocity versus bow inclination. The grey areas indicate the string-crossing areas, and the center of the string crossings are indicated by the vertical dashed lines. The thick solid lines show the grand average curves per condition (blue = down bow, red = up bow), and the thin lines indicate the individual averages. The columns (panels A/B and C/D) represent the two string combinations (E-A and A–D, respectively), and the rows (panels A/C and B/D) represent dynamic level (forte and piano, respectively).

**Figure 7:**
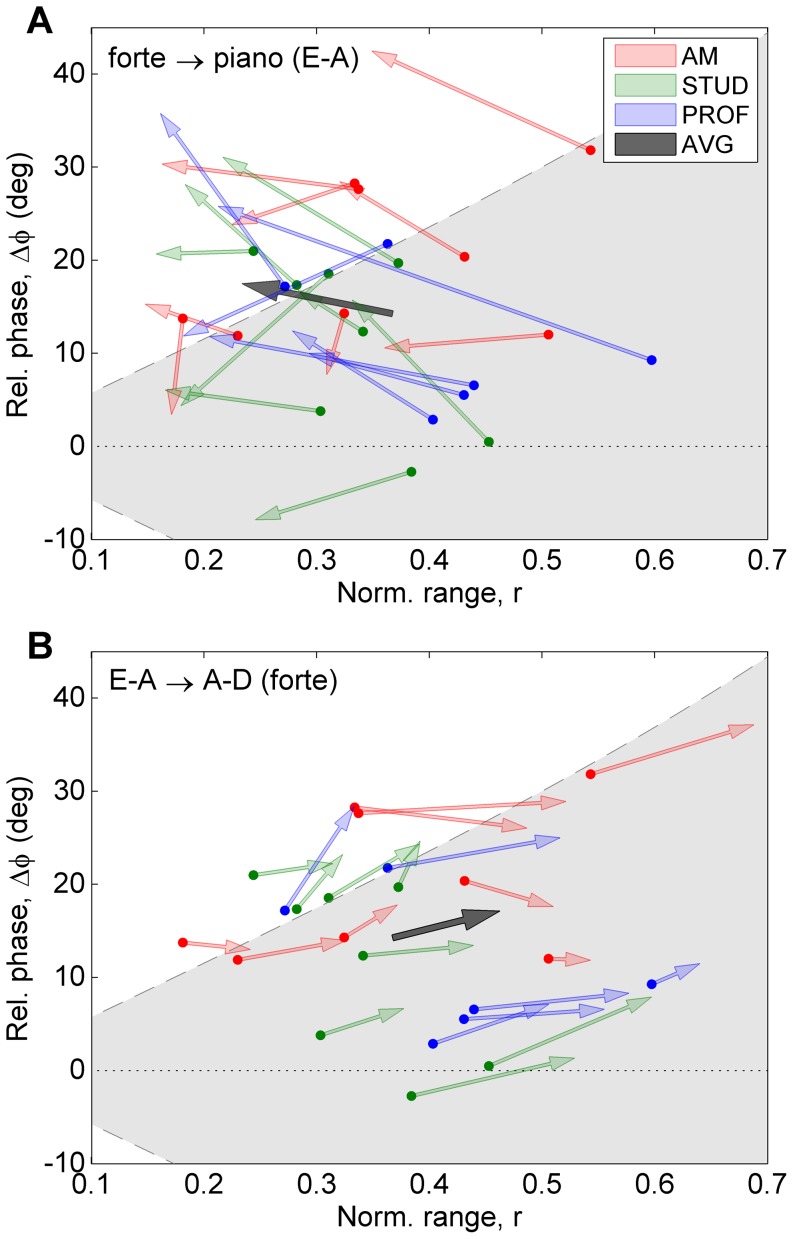
Comparisons between conditions in the dynamic-level block. The plots give an overview of how the individual participants adapted the main coordination parameters between selected clockwise-pattern conditions in the 2D coordination space. Skill level is indicated by color (see legend). The dark grey arrow shows the average across all participants. The panels show (A) change from forte to piano (at the E-A string combination), and (B) the change from string combination E-A to A–D (at forte dynamic level).

The independent variables in the dynamic-level conditions were dynamic level (cf., movement amplitude), string combination (cf., physical constraints of the strings), and level of expertise. The analyses were performed on a selection of conditions from the dynamic-level condition block, including only clockwise circular patterns (CW). A 2×2×3 mixed-model design was used with two within-subject factors [“dynamic level” (two levels: forte, piano), and “string combination” (two levels: E-A, A–D)], and one between-subject factor [“level of expertise” (three levels: amateur, student, professional)]. ANOVAs were performed separately for six dependent variables: relative phase (mean and stdev), normalized range (mean and stdev), and inclination offset (mean and stdev). Mean values (per participant and condition) reflect coordination strategy, whereas standard deviations reflect stability of performance. Figure 6 presents an overview of the coordination patterns (bow velocity vs. bow inclination) per condition. Figure 7 gives an indication of the changes between selected conditions projected onto the 2D coordination parameters space (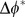 vs. 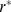). The latter graph shows an overview of the individual participants (colored arrows) as well as the general trend (grey arrows), providing insight into the degree of coherence of the participants' behavior.

#### Relative phase (mean)

There was one significant main effect of “string combination” [F(1,19) = 13.1; p<0.05; 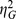 = 0.013 (very small)]. There were no significant interactions. Post-hoc comparison revealed that relative phase was higher at string combination A–D (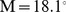) compared to E-A (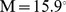). The increase of 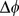 and the relatively good agreement between individual participants with respect to this can also be observed in Figure 7B (only showing forte level).

#### Normalized range (mean)

There were two significant main effects of “dynamic level” [F(1,19) = 85.6; p<0.001; 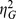 = 0.38 (large)] and “string combination” [F(1,19) = 206.3; p<0.001; 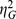 = 0.22 (medium)]. There were no significant interactions. Post-hoc comparison revealed that 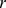 was larger at forte (M = 0.41) compared to piano (M = 0.28), and that 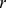 was larger at string combination A–D (M = 0.39) compared to E-A (M = 0.30).

**Figure 8:**
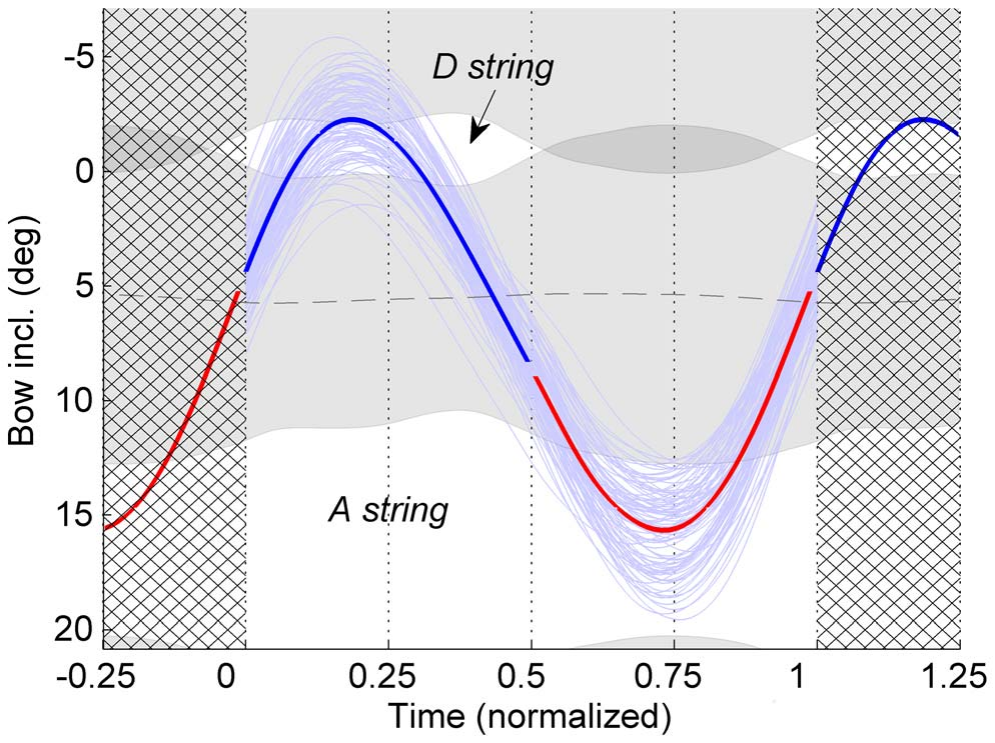
Example of range constraints in an individual (professional) participant. Bow inclination versus time (normalized with respect to cycle duration) per individual cycle (thin lines) and averaged across cycles (thick colored line). The grey areas indicate the averaged string-crossing areas as a function of time. Bowing direction is indicated by the color of the curve (blue = down bow, red = up bow).

The difference between the conditions with respect to inclination amplitude and string-crossing range is illuminated in Figure 6, showing the grand average of the bow-velocity vs. bow-inclination curves across participants. At forte level (panels A and C) the string-crossing area was about a factor two larger compared to piano level, which could mainly be attributed to an increase of bow force. The inclination amplitude was also higher at forte level, but less than proportional with respect to the string-crossing area, explaining the effect of “dynamic level” on normalized range. Furthermore, it can be observed that the angular range of the D string was rather limited at forte level (panel C), so that contact with the next neighboring string became harder to avoid. This was especially the case in one of the professional participants (PROF3), who used a rather high bow force (about 1.5 N), leaving a very limited space for the D string between the two string-crossing areas (see Figure 8). Accordingly, the inclination amplitude was restricted, leading to a larger value of 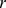.

#### Inclination offset (mean)

There were two significant main effects of “dynamic level” [F(1,19) = 5.8; p<0.05; 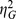 = 0.04 (small)] and “string combination” [F(1,19) = 39.8; p<0.001; 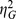 = 0.39 (large)]. There were no significant interactions. Post-hoc comparison revealed that inclination offset was approximately zero at the A–D string combination at both dynamic levels. At the E-A string combination, the inclination offset was larger at piano (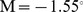) compared to forte (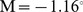). The negative sign indicates that the inclination offset was towards the A string. It should be noted that at piano level, the string-crossing area was small compared to forte so that the effect of the offset on the asymmetry with respect to the string crossing became even larger. In this case the inclination offset at piano level caused that, on average, the bow change from up to down bow took place well outside the string-crossing range, as can be seen in Figure 6B.

#### Relative phase (stdev)

There was one significant main effect of “dynamic level” [F(1,19) = 7.9; p<0.05; 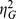 = 0.07 (small)]. There were no significant interactions. Post-hoc comparison revealed that the standard deviation of relative phase was larger at piano (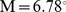) compared to forte level (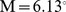).

#### Normalized range (stdev)

There were two significant main effects of “dynamic level” [F(1,19) = 10.5; p<0.01; 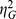 = 0.08 (small)] and “string combination” [F(1,19) = 55.0; p<0.001; 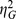 = 0.14 (medium)]. There were no significant interactions. Post-hoc comparison revealed that the standard deviation of 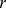 was larger at forte (M = 0.070) compared to piano level (M = 0.059), and that it was larger at the A–D string combination (M = 0.072) compared to E-A (M = 0.057).

The finding that the standard deviation of 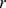 was larger at forte compared to piano level was surprising at first thought since it was expected that the stability of the movements would be larger at higher amplitudes of movement. However, it should be realized that normalized range is calculated as the division of the absolute string-crossing width with peak-to-peak inclination extent. The averages of both are larger at forte level, and their respective variances as well. Consequently, the variance of their quotient is larger at forte level. Considering the coefficients of variance of the absolute string-crossing range and the inclination extent, it was found that both were significantly smaller at forte compared to piano, indicating that the relative stability of both components was higher at forte level [“CV of string-crossing width”: F(1,19) = 13.8; p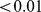; 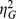 = 0.14 (medium), and “CV of inclination extent”: F(1,19) = 28.2; p<0.001; 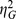 = 0.21 (medium)].

#### Inclination offset (stdev)

There were two significant main effects of “level of expertise” [F(2,19) = 4.3; p<0.05; 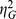 = 0.18 (medium)] and “dynamic level” [F(1,19) = 9.2; p<0.01; 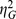 = 0.07 (small)]. There were no significant interactions. Post-hoc comparison revealed a significant difference between amateurs (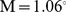) and students (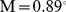), indicating that inclination offset was most stable in the student group, followed by professionals (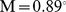) and amateurs. Concerning “dynamic level”, the standard deviation of inclination offset was larger at forte (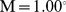) compared to piano level (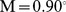).

Similarly as for normalized range above, it can be argued that the increase of the standard deviation at forte level should be considered in relation with the increased movement amplitude. By taking inclination extent as a reference, it was found that the coefficient of variation of inclination offset was smaller at forte compared to piano level, indicating a higher relative stability at forte level [F(1,19) = 72.4; p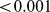; 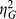 = 0.41 (large)].

### Tempo, pattern and expertise

The independent variables in the tempo conditions were tempo (cf., movement frequency), pattern (cf., level of difficulty), and level of expertise. A 4×3×3 mixed-model design was used with two within-subject factors [“tempo” (four levels: 54, 72, 92, 112 bpm), and “pattern” (three levels: CW, ACW, Fo8)], and one between-subject factor [“level of expertise” (three levels: amateur, student, professional)]. ANOVAs were performed separately for six dependent variables: relative phase (mean and stdev), normalized range (mean and stdev), and inclination offset (mean and stdev). Mean values (per participant and condition) reflect coordination strategy, whereas standard deviations reflect stability of performance. One of the amateur participants (AM1) had to be omitted from the analyses because pattern ACW was missing, reducing the group size of amateurs to seven participants.

Since the amplitude-based estimation of 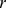 is not defined for pattern Fo8, the alternative time-domain estimate 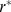 was used. Furthermore, the relative Hilbert phase of Fo8 was not always straightforward to interpret, especially at the lowest tempi. However, the time-domain estimate of relative phase 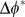 was not considered a proper alternative in all cases, especially when considering stability aspects, since it might be contaminated by fluctuations of inclination offset (for an explanation, see “Materials and Methods: Data analysis”). Therefore, both measures of relative phase will be considered, taking into account their respective strengths and weaknesses.

**Figure 9:**
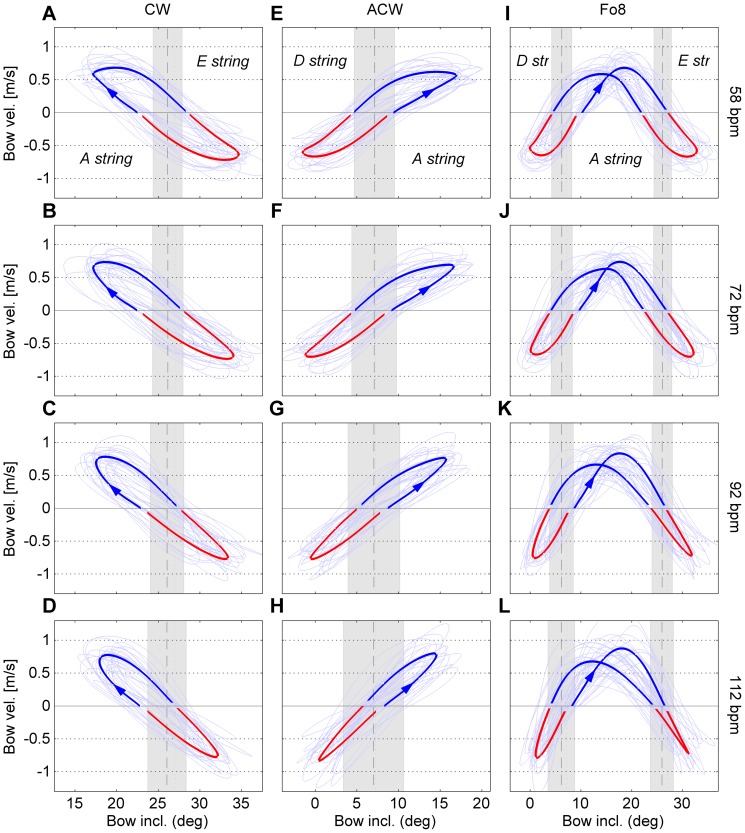
Coordination patterns of all twelve conditions in the tempo block. The columns (panels A–D, E–H, I–L) represent the bowing patterns (CW, ACW and Fo8, respectively). The rows (panels A/E/I, B/F/J, etc.) represent tempo (58, 72, 92 and 112 bpm, respectively). For a detailed explanation of the graphs, see Figure 6.

**Figure 10:**
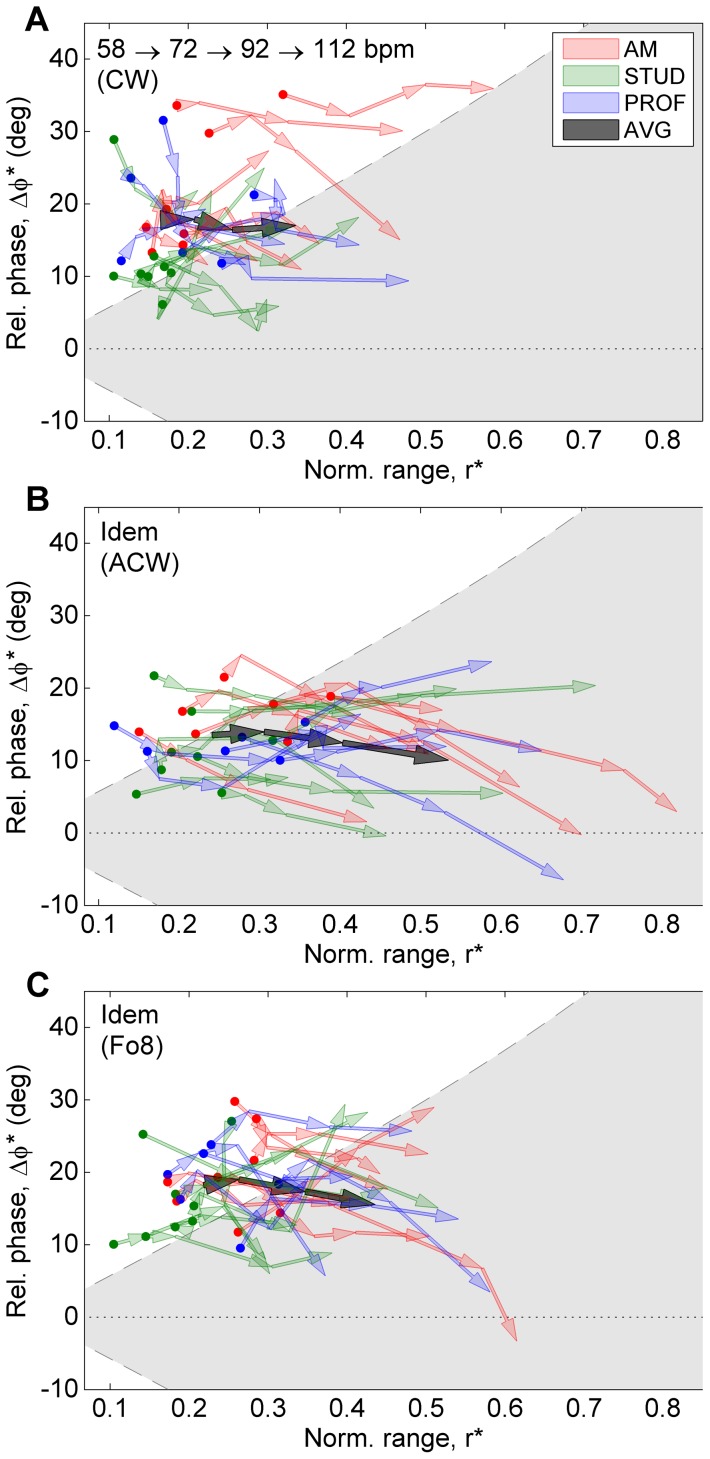
Comparisons between conditions in the tempo block. The panels show the adaptation of the coordination parameters from the slowest to the fastest tempo in (A) pattern CW, (B) pattern ACW, and (C) pattern Fo8. For a detailed explanation of the graphs, see Figure 7.

Figure 9 presents an overview of the coordination patterns (bow velocity vs. bow inclination) per condition. Figure 10 gives an indication of the changes between selected conditions projected onto the 2D coordination parameters space (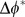 vs. 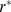).

#### Relative phase (mean)

Given the problems with the estimation of the relative Hilbert phase in pattern Fo8 at low tempi, the time-domain estimate of relative phase 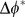 was chosen as the dependent variable. There was one significant main effect of “pattern” [F(2,36) = 11.5; p<0.001; 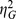 = 0.14 (medium)]. An initially weakly significant main effect of tempo became non-significant after sphericity correction (Greenhouse-Geisser), and no apparent trend with regard to tempo could be observed. There were no significant interactions. Post-hoc comparison revealed that relative phase was largest in pattern Fo8 (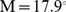), followed by CW (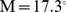) and ACW (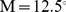), the contrast between CW and ACW just failing significance (p = 0.052, Holm-adjusted).

An ANOVA with the Hilbert-based estimate 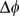 as dependent variable, excluding pattern Fo8 from the analysis, yielded comparable results (with average values of CW and ACW of 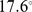 and 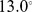, respectively), confirming the observed effects in 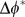 described above.

#### Normalized range (mean)

There were two significant main effects of “tempo” [F(3,54) = 254.6; p<0.001; 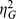 = 0.53 (large)] and “pattern” [F(2,36) = 43.1; p<0.001; 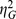 = 0.31 (large)]. Post-hoc comparison showed a clear increase of 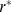 with increasing tempo, which can be clearly seen in Figure 10. Closer inspection of Figure 9 reveals that this increase can be attributed to both an increase of the string-crossing area and a decrease of inclination amplitude with increasing tempo. With regard to “pattern”, 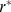 was largest in pattern ACW (M = 0.37), followed by Fo8 (M = 0.31) and CW (M = 0.24), the difference between ACW and CW being significant. Furthermore, there was a significant interaction of “tempo x pattern” [F(6,108) = 22.3; p<0.001; 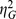 = 0.07 (small)]. Post-hoc analysis revealed that in pattern ACW, 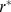 increased more strongly with tempo than in the other two patterns, as can be clearly seen in Fig. 10.

#### Inclination offset (mean)

There was one significant main effect of “pattern” [F(2,36) = 6.9; p<0.01; 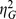 = 0.13 (medium)]. Post-hoc comparison revealed that the inclination offset was largest in pattern CW (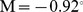), followed by Fo8 (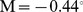) and ACW (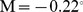). However, it should be noted that the patterns involved different string combinations, which might form a confounding factor. Pattern CW was played on the E and the A strings, whereas pattern ACW was played on the A and the D strings, which might explain the observed difference (see influence of inclination offset (mean) in the dynamic-level conditions above). Pattern Fo8 involves three strings (E, A and D), making direct comparison with the other two patterns with respect to inclination offset difficult.

#### Relative phase (stdev)

For the analysis of the stability (stdev) of relative phase, the choice for the estimation method of the dependent variable (Hilbert- or time-domain-based) was not obvious since both contain weaknesses, which might influence the outcome. Therefore, ANOVAs of both were performed, and the observed effects were interpreted trying to separate true effects from possible artifacts.

In the ANOVA with the standard deviation of 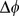 (Hilbert-based estimate) as dependent variable, there were two significant main effects of “level of expertise” [F(2,18) = 7.6; p<0.01; 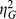 = 0.15 (medium)] and “pattern” [F(2,36) = 158.7; p<0.001; 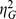 = 0.77 (large)]. There were no significant interactions. Post-hoc comparison revealed that the amateur group showed the largest standard deviation (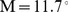), followed by the professional group (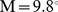) and the student group (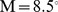); however, none of the contrasts reached a significant level. The standard deviation of 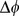 in pattern Fo8 (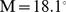) was significantly larger than that in patterns ACW (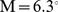) and CW (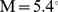). However, it should be noted that the variance in pattern Fo8 was likely overestimated, especially at lower tempi.

As a check, an ANOVA was performed with the standard deviation of 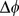 as dependent variable, leaving out pattern Fo8 from the analysis. This yielded a comparable main effect of “level of expertise” [F(2,18) = 13.0; p<0.001; 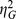 = 0.30 (large)]. There was still a main effect of “pattern” [F(1,18) = 15.3; p<0.01: 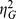 = 0.18 (medium)] and an additional main effect of “tempo” [F(3,54) = 15.6; p<0.001; 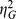 = 0.15 (medium)]. In addition, there was a significant interaction of “tempo x pattern” [F(3,54) = 5.3; p<0.01; 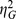 = 0.06 (small)]. Post-hoc analysis revealed that the standard deviation of 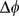 increased with increasing tempo, more strongly so in pattern ACW compared to pattern CW. The standard deviation of 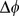 in pattern ACW was larger than in pattern CW (as above).

Finally, the ANOVA with the standard deviation of 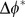 as dependent variable yielded three significant main effects of “level of expertise” [F(2,18) = 25.4; p<0.001; 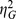 = 0.47 (large)], “tempo” [F(3,54) = 103.2; p<0.001; 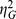 = 0.39 (large)], and “pattern” [F(2,36) = 52.4; p<0.001; 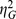 = 0.51 (large)]. There was one significant interaction of “tempo x pattern” [F(6,108) = 10.43; p<0.001; 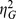 = 0.11 (small)]. Post-hoc comparison revealed that the standard deviation of 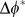 was largest for the amateur group (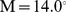), followed by the professional (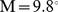) and the student group (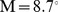), the contrast between the amateur and the student group just failing significance (p = 0.062, Holm-adjusted). With regard to “tempo”, the standard deviation of 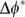 showed a clear increase with increasing tempo in all patterns. However, it should be noted that this is probably partly due to an artifact inherent in the estimation method (see “Materials and Methods” for details). With regard to “pattern”, the standard deviation of 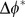 was largest in pattern Fo8 (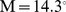), followed by ACW (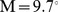) and CW (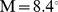). In addition, the patterns showed a different dependency of tempo (interaction “tempo x pattern”), the standard deviation of 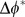 in pattern Fo8 showing the largest increase with increasing tempo, followed by ACW and CW.

Putting the pieces of the puzzle together, we can list the following dependencies of the stability of relative phase. There was a clear dependence on “level of expertise” (medium-large effect), the student group showing the highest stability, followed closely by the professional group, and at a larger distance by the amateur group. Stability seemed to be rather independent of “tempo”. Possibly, stability might show a larger dependence of tempo with increasing level of difficulty of the pattern as indicated by the significant interactions (small effect), the stability of Fo8 decreasing most strongly with increasing tempo, followed by ACW and CW. With regard to “pattern” (medium-large effect) the results indicate that stability decreased with increasing level of difficulty of the pattern, pattern CW being most stable, followed closely by ACW and at a larger distance by Fo8.

**Normalized range (stdev)** There were three significant main effects of “level of expertise” [F(2,18) = 3.7; p<0.05; 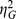 = 0.17 (medium)], “tempo” [F(3,54) = 70.0; p<0.001; 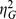 = 0.29 (large)], and “pattern” [F(2,36) = 29.1; p<0.001; 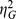 = 0.30 (large)]. There were no significant interactions. Post-hoc comparison revealed that the standard deviation of 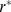 was smallest for the student group (M = 0.050) compared to the professional (M = 0.066) and the amateur group (M = 0.069); however, none of the contrasts reached a significant level. There was a clear increase with increasing tempo. Furthermore, pattern Fo8 showed the largest standard deviation (M = 0.075), followed by ACW (M = 0.063) and CW (M = 0.044), the contrast between Fo8 and CW reaching a significant level (p<0.001, Holm-adjusted).

An ANOVA with the standard deviation of the amplitude-based estimate of normalized range (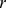) as dependent variable, excluding pattern Fo8 from the analysis, yielded two significant main effects of “tempo” [F(3,54) = 88.2; p<0.001; 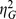 = 0.38 (large)] and “pattern” [F(1,18) = 59.2; p<0.001; 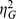 = 0.30 (large)], as well as two significant interactions of “level of expertise x tempo” [F(6,54) = 2.5; p<0.05; 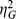 = 0.03 (small)] and “tempo x pattern” [F(3,54) = 16.3; p<0.001; 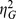 = 0.10 (small)]. Post-hoc analysis revealed an increase of the standard deviation of 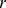 with increasing tempo. The standard deviation in pattern ACW (M = 0.072) was larger than that in CW (M = 0.045). With regard to the interactions, the amateur group showed a stronger dependency on tempo than the other two groups, and pattern ACW was more strongly dependent on tempo than CW.

Taken together, we can list the following dependencies of the stability of normalized range. With regard to “level of expertise” (medium effect), the student group seemed to be most stable, however, the differences between groups were rather small. The stability decreased with increasing tempo (large main effect), possibly more so for the more difficult patterns (small interaction effect). Furthermore, stability decreased with increasing level of difficulty of the pattern (large main effect).

#### Inclination offset (stdev)

There were three significant main effects of “level of expertise” [F(2,18) = 6.2; p<0.01; 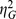 = 0.20 (medium)], “tempo” [F(3,54) = ; p<0.01; 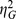 = 0.03 (small)], and “pattern” [F(2,36) = 11.7; p<0.001; 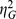 = 0.14 (medium)]. There were no significant interactions. Post-hoc comparison revealed that the standard deviation of *θ*_offset_ was highest in the amateur group (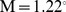), followed by the professional group (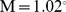) and the student group (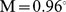). The dependence on tempo was not very clear; a slight increase with increasing tempo could be observed. With regard to “pattern”, the standard deviation of *θ*_offset_ was highest in pattern Fo8, followed by ACW and CW; however, none of the contrasts reached a significant level.

## Discussion and Conclusions

### General coordination behavior

It could be confirmed that the general coordination behavior was consistent across participants and conditions, characterized by a phase lead of bow inclination relative to bow velocity of about 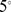
–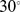, as well as an approximate upper limit of 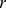 of about 0.6, in agreement with earlier findings related to the perception and production of fast repetitive bowing patterns [3]–[5]. The distributions of the main coordination parameters 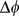 and 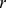 was strikingly similar to those found in a perception study of similar bowing patterns controlling a virtual violin [5], [79], providing a strong indication that the observed motor behavior has been optimized with regard to the auditory outcome.

### Coordination strategies and stability

In the following discussion general aspects of the influence of the experimental conditions on coordination behavior will be considered. It should be stressed that the indicated relations do not necessarily apply at an individual level, given the large inter-individual differences with regard to coordination strategy and the use of other bowing parameters, such as bow force and bow-bridge distance. As a result, the constraints associated with the experimental conditions were not always the same for all individuals, in particular not those related to string crossings, which are heavily dependent on bow force and bow-bridge distance [61]. Figures 7 and 10 provide a graphical impression of the coherence of the individuals' strategies and the way they adapted to different conditions (colored arrows) in comparison with the general trends (dark grey arrows).

With regard to *level of expertise*, the results did not indicate a significant distinction at group level regarding coordination strategy. This is against the expectation that expert performance would show a larger inter-individual agreement compared to less skilled performers. Furthermore, there were no signs that skill level had an influence on the ability to adapt to increasingly difficult performance situations. For example, in Figure 10B and C there is no systematic distinction between groups in how coordination strategies are altered with increasing tempo. It should be noted, however, that the performance conditions within the current experiment were not highly extreme. For example, the highest tempo (112 bpm) is still quite moderate, and it cannot be excluded that the more skilled participants would be able to perform the patterns at higher tempi at which the less skilled ones would need to give up.

The results did indicate a significant difference between groups with respect to stability of performance, at least in the tempo conditions. The student group tended to be most stable, followed closely by the professional group, and at a larger distance by the amateur group. This is roughly in agreement with the expectation that more skilled performers show a higher stability of performance. In the dynamic-level conditions the differences were less clear. A possible explanation for the latter is that in the dynamic-level conditions, tempo was not controlled for, allowing participants to choose a tempo at which they felt comfortable. Indeed the amateurs (104 bpm, range 81–126 bpm) performed the dynamic-level conditions on average slower than the students (109 bpm, range 92–128 bpm) and the professionals (123 bpm, range: 111–137 bpm).

The limited effects of expertise level might be due to a ceiling effect; first, the amateur participants in this experiment were all rather advanced, and second, the tasks were relatively undemanding. Apparently, this type of coordination behavior should be considered as a rather basic skill, and a cross-sectional study of its development would require inclusion of participants at earlier stages of progress. For example, in other studies of violin and cello performance, clear distinctions were found between novices and more advanced players with regard to basic performance skills [19], [20]. Furthermore, the finding that the student group tended to be more stable than the professional group, even though the differences were small, is noteworthy. It might well be the case that students at this level have reached a mature level with regard to motor performance, and that professionals do not distinguish themselves by a further perfection of motor performance, but rather by excellence in other qualities such as expressiveness and stage presence, which were not addressed in the current experimental design. Another possible explanation is that the students on average spent more time on individual practicing at the time of the experiment.

As a final remark, the large inter-individual variation of coordination strategies puts into question the existence of a well-defined optimal coordination strategy. A preliminary analysis in which expert ratings of the recorded audio were projected onto the 2D coordination space (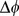 vs. 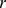) did not reveal any systematic relationship between the coordination parameters and the perceived quality of performance [80]. These findings indicate that the acoustical constraints with regard to the coordination of bow changes and string crossings are rather loose, suggesting the existence of a certain acceptance area in the 2D coordination space. The latter is supported by findings from a complementary study, in which a set of perceptual criteria was derived with respect to acoustical features of simulated violin performances using a gesture-controlled synthesizer [62]. The acceptance area might give performers the possibility to control the perceptual quality of the note transitions in accordance to personal taste or expressive purposes [80].

There were clear effects of the *level of difficulty* of the patterns, in agreement with the expectations. All three stability measures (standard deviations of phase, range and inclination offset) showed a clear dependence of pattern. The clockwise pattern (CW) was most stable, followed by the anti-clockwise pattern (ACW) and the figure-of-eight pattern (Fo8). Furthermore, there was a significant interaction between pattern and tempo with regard to phase and range stability; the more difficult the pattern, the larger the decrease of stability with increasing tempo. Finally, there was a clear effect of pattern on the main coordination parameters phase and range. Compared to the other two patterns, the ACW pattern showed 1) a small relative phase, 2) a large range, and 3) a stronger increase of range with increasing tempo. This might indicate that it was difficult to achieve a favorable coordination strategy in this pattern. This can also be seen in Figure 10B, where some participants ended up at exceptionally large range and small phase values at the highest tempo.

With regard to tempo (movement frequency), it was shown that the relative phase was rather constant. The normalized range showed a clear increase with increasing tempo, which could be attributed to 1) a broadening of the string-crossing range due to an increase of bow force, and 2) a decrease of inclination amplitude (see Figure 9). The dependence of normalized range on tempo might be partly explained by the physical conditions for producing a good tone. The increase of bow force likely served as an adaptation to the increase of acceleration at the bow changes in order to guarantee a proper speaking of the strings, in accordance with Guettler [8], who showed that there exists a proportional relationship between bow force and bow acceleration for producing a good note start. (The increase of acceleration with increasing tempo is evident from Figure 9 by the fact that the velocity amplitude was rather constant or even increased with increasing tempo.) With regard to inclination amplitude, there is not such a straightforward explanation. One could expect that performers might reduce the amplitude with increasing tempo since maintaining a constant amplitude requires an increase of muscle effort. However, the duration of the string crossing is affected by tempo, which in its turn affects the physical conditions of the string transitions, as well as the perceptual quality criteria. The relation between normalized range and tempo can therefore be expected to be the result of a combination of several constraints and criteria, making it hard to resolve.

The variability of relative phase was found to be rather independent of tempo, at least for the easiest CW pattern (see discussion of level of difficulty above). This indicates a robust coupling between the two movement components with regard to their phasing. The variability of range increased with increasing tempo, indicating that the control of inclination amplitude and/or bow force became increasingly difficult.

With regard to dynamic level (movement amplitude), relative phase was found to be rather constant. Normalized range was larger at forte compared to piano level, due to an increased width of the string-crossing range. The inclination amplitude was also increased, but not in proportion. This might be partly due to geometrical constraints of the instrument since at high bow force it becomes harder to avoid contact with the next neighboring strings (see Figure 8 for a clear example of this). With regard to stability only small effects were found. Relative phase showed a higher variability at forte level, indicating a positive influence of movement amplitude on coordination stability. The standard deviations of normalized range and inclination offset were both larger at forte compared to piano level; however, the implications with regard to stability should be carefully considered taking the increased amplitude of the movements into account. Concerning normalized range it was shown that the coefficients of variance of absolute string-crossing width and peak-to-peak inclination extent were smaller at forte level, indicating a higher relative stability of both components. Along similar lines, it was shown that the relative stability of inclination offset was higher at forte compared to piano level.

Finally, a marked effect of string combination on the inclination offset was found. On the E-A string combination the center of the inclination movement was shifted towards the lower A string, whereas on the A–D string combination the center of the inclination movement coincided almost perfectly with the center of the string crossing. Interestingly, the inter-individual agreement was rather high. This behavior can most likely be attributed to the playing properties of the strings; however, an unequivocal explanation is hard to provide. One possibility could be that it is due to an asymmetry of the characteristic impedances of the strings involved. However, in that case one would rather expect that the inclination offset would be larger on the A–D string combination since their characteristic impedances differ more than those of the E and the A string (the characteristic impedances of the E, A and D string used in this experiment were 0.18, 0.2 and 0.25 kg/s, respectively). Another possibility is that the players tried to avoid too much emphasis on the open E string, which is known to have a more penetrating sound quality in comparison with the other strings. Not much is known about how players adapt their bowing to different types of strings, especially not when played in combination, for example in double stops or complex bowing patterns involving multiple strings. More systematic experimentation would be needed to clarify this.

### Coordination and perception-action coupling

The bowing patterns in the current study feature a highly specific phase relation and a highly stable performance. The circular patterns CW and ACW feature a relative phase of 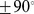 in the spatial domain, with an additional phase shift of about 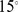, so that they can be described as slanted ellipses [21]. The figure-of-eight (Fo8) pattern is characterized by a 2∶1 frequency relation and a similar phase shift, yielding a slightly deformed figure-of-eight shape in the spatial domain.

In the context of laboratory studies of coordination behavior (e.g., intralimb, or intrasegmental coordination in circle drawing [21], [22] and other types of arm movements [25], as well as bimanual coordination [29], [31]), the bowing movements in the current study possess some distinguishing features. First, they are performed at a relatively high movement frequency, in this study up to about 4 Hz, but they can be performed even faster. Second, they display a relatively high stability with standard deviations of relative phase down to about 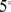, compared to typical values of 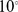 and higher reported in literature (e.g., [22], [32]–[35], [63]). Finally, the specific phase relations observed in the current study, characterized by phase shifts in the range of 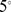
–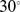, form an interesting contrast compared to the general focus on in-phase and anti-phase behavior in scientific studies of coordination, explained in terms the dynamics of the motor system [26], [29], [63], [81] or perception-action coupling [25], [30], [32], [33], [37], [82].

Several studies have demonstrated the influence of visual information (in the form of a stimulus, or visual feedback) on the stability or other qualities of coordination [82], [83], and on the ability to learn unusual coordination patterns involving phase shifts [25], [32], [34], [84], [85], specific frequency ratios [35], or both [33]. In case of violin bowing, the primary source of feedback is the sound of the instrument, which has a direct relationship with the performer's action in a complex and nonlinear manner [6]–[10]. The findings of the current study, in combination with findings from a complementary perceptual study [5], strongly suggest that feedback in the form of a complex auditory stream can play a crucial role in the formation of specific coordination patterns.

### Future directions

In the near future a follow-up experiment is planned, in which an altered auditory feedback paradigm will be employed to investigate direct influences of auditory feedback on the coordination behavior in fast repetitive bowing patterns. With that experiment we intend to shed further light onto the possibly emergent character of the observed coordination behavior by studying how performers adapt to artificially induced perturbations.

The analyses presented in the current paper were limited to movements of the bow relative to the violin. Further analyses of the full body motion-capture data, in particular that of the right arm, need to be performed in order to shed light onto kinematic and kinetic aspects in the production of fast repetitive bowing patterns. Such an analysis could provide further explanations of biomechanic functioning and expertise-related differences in performance, as demonstrated in piano performance [86], and other bowing techniques in violin and cello performance [11], [12], [16], [17].

Ultimately, we aim to apply the methods developed in the context of this study for analysis of coordination impairments in musician patients suffering from pathological movement disorders, such as action-induced tremor and focal dystonia.

As a concluding remark, we propose that bowed-string instrument performance can offer an interesting model for studying auditory- and sensory-motor control in complex repetitive and serial movement patterns as a complement to artificial tasks in controlled laboratory experiments.

## Supporting Information

Video S1 **Video example of clockwise circular bowing pattern (CW).** Animated display of a performance by participant STUD5 (tempo 92 bpm). The two left panels show the movements of the bow relative to the violin projected from above (top-left) and behind (bottom-left). The solid black line shows the position of the bow-hair ribbon from the frog to the tip. The curves show the movement trajectory of the bow frog close to the hand of the player. The light background colors in the bottom-left panel indicate the different strings (fixed angels), and bow force is indicated by the color of the movement trajectory. The top-right panel shows bow inclination versus time. The light background colors indicate the different strings, showing the time-varying angular areas of the strings, including a correction for finger stopping (left hand) and the estimated string-crossing areas (overlapping colors). The bottom-right panel shows bow inclination versus bow velocity, most clearly revealing the relative phase between the two main movement components.
(MP4)

Video S2 **Video example of anti-clockwise circular bowing pattern (ACW).** Animated display of a performance by participant STUD5 (tempo 92 bpm). For an explanation of the panels, see Video S1.
(MP4)

Video S3 **Video example of figure-of-eight bowing pattern (Fo8).** Animated display of a performance by participant STUD5 (tempo 92 bpm). For an explanation of the panels, see Video S1.
(MP4)

Dataset S1 **Dataset documentation.** PDF document, describing the dataset files (Dataset S2 and S3).
(PDF)

Dataset S2 **Participant information.** Table (tab-separated .csv) with participant information, including the measures of expertise.
(CSV)

Dataset S3 **Features per participant and condition.** Table (tab-separated .csv) with means and standard deviations of extracted features per participant and condition.
(CSV)
