# Supplementary material for: Coordination in Fast Repetitive Violin-Bowing Patterns
Source: PLoS One. 2014 Sep 10;9(9):e106615. doi: 10.1371/journal.pone.0106615 (PMC4160185; doi:10.1371/journal.pone.0106615)
Supplement: Dataset S1 — Dataset documentation. PDF document, describing the dataset files (Dataset S2 and S3). (PDF) [file pone.0106615.s004.pdf]

Dataset documentation

File format

The dataset files are formatted as tab-separated .csv files, with the first row as a header. They can be opened in standard office software (MS Excel, LibreOffice, OpenOffice).[1]

In R the datasets can be opened using the `read.table` function (replacing “`dataset.csv`” by the correct file name):

```
> dat <- read.table("dataset.csv", header=TRUE, sep="\t")
```

In Matlab the datasets can be opened using the `dataset` function (Statistics Toolbox required):

```
>> dat = dataset('file', 'dataset.csv');
```

Dataset S5: Participant information

Column legend:

- group: classification of participants according to level of expertise (**AM**ateur, **STUD**ent, **PROF**essional)
- subject: participant ID number
- sex: sex of participants (**m**ale/**f**emale)
- length: length of the participants (cm)
- weight: weight of the participants (kg)
- age: age of the participants (years)
- starting_age: age at which the participants started playing the violin (years)
- handedness_index: handedness index value in percent from -100 (left handed) to 100 (right handed)
- handedness: handedness classification (**r**ight/**l**eft)
- omsi_p: OMSI-p value from the Ollen Musical Sophistication Index questionnaire from 0 (low level of musical sophistication) to 1 (high level of musical sophistication).
- hours_tot: total cumulative time spent on deliberate practice on the violin (hours).
- hours_12: cumulative practice hours at the age of 12
- hours_21: cumulative practice hours at the age of 21
- current_hours_per_day: hours per day currently spent on deliberate practice on the violin

1 Make sure that the regional settings comply to the use of a . (dot) as a decimal separator.

Dataset S6: Features per participant and condition

Meta information (columns 1-7):

- group: classification of participants according to level of expertise (**AM**ateur, **STUD**ent, **PROF**essional)
- subject: participant ID number
- condition_id: unique condition identifier (integer number)
- block: condition block (dynamics: dynamic level conditions; tempo: tempo conditions; preludio: fragments of the Preludium of the third sonata for solo violin by J.S. Bach)
- pattern: bowing pattern (CW: clockwise circular; ACW: anti-clockwise circular; Fo8: figure-of-eight)
- tempo: nominal tempo of a quarter note (bpm), only accurate in the tempo conditions (performance with metronome)
- dynamic_level: instructed dynamic level (most relevant to the dynamic level conditions)

Feature data. Features were extracted from the processed motion-capture data at each bow change. The feature table contains the means (suffix _av) and standard deviations (suffix _sd) of the features per participant and condition.

Selected features (columns 8-30):

- N_tot: total number of bow changes used for calculation of the means (_av) and standard deviations (_sd)
- phase_rel_hilbert: relative phase based on a Hilbert transform (deg)
- phase_rel_alt: relative phase based on alternative time-domain estimation (deg)
- range_rel_tc: normalized range (amplitude-based estimation at the center of the string crossing)
- range_rel_alt: normalized range (alternative time-domain estimation)
- incl_offset: inclination offset
- string_crossing_extent: width of string-crossing area (deg)
- incl_extent: peak-to-peak extent of bow inclination movement (deg)
- bow_pos: position (mm) in the length direction of the bow at which the patterns were performed (center of bow displacement)
- bow_vel_ampl: bow velocity amplitude (peak bow velocity, mm/s)
- bow_force: normal force exerted by the bow on the string (N)
- beta: bow-bridge distance, normalized with respect to the speaking length of the string
